# Granulocyte colony stimulating factor attenuates inflammation in a mouse model of amyotrophic lateral sclerosis

**DOI:** 10.1186/1742-2094-8-74

**Published:** 2011-06-28

**Authors:** Eveliina Pollari, Ekaterina Savchenko, Merja Jaronen, Katja Kanninen, Tarja Malm, Sara Wojciechowski, Toni Ahtoniemi, Gundars Goldsteins, Raisa Giniatullina, Rashid Giniatullin, Jari Koistinaho, Johanna Magga

**Affiliations:** 1 A.I. Virtanen Institute for Molecular Sciences, University of Eastern Finland, Kuopio, Finland; 2 Medeia Therapeutics Ltd, Kuopio, Finland; 3 Department of Oncology, Kuopio University Hospital, Kuopio, Finland; 4 Institute of Biomedicine, University of Oulu, Oulu, Finland

**Keywords:** Amyotrophic lateral sclerosis, GCSF, pegfilgrastim, inflammation, monocytes, cytokines

## Abstract

**Background:**

Granulocyte colony stimulating factor (GCSF) is protective in animal models of various neurodegenerative diseases. We investigated whether pegfilgrastim, GCSF with sustained action, is protective in a mouse model of amyotrophic lateral sclerosis (ALS). ALS is a fatal neurodegenerative disease with manifestations of upper and lower motoneuron death and muscle atrophy accompanied by inflammation in the CNS and periphery.

**Methods:**

Human mutant G93A superoxide dismutase (SOD1) ALS mice were treated with pegfilgrastim starting at the presymptomatic stage and continued until the end stage. After long-term pegfilgrastim treatment, the inflammation status was defined in the spinal cord and peripheral tissues including hematopoietic organs and muscle. The effect of GCSF on spinal cord neuron survival and microglia, bone marrow and spleen monocyte activation was assessed *in vitro*.

**Results:**

Long-term pegfilgrastim treatment prolonged mutant SOD1 mice survival and attenuated both astro- and microgliosis in the spinal cord. Pegfilgrastim in SOD1 mice modulated the inflammatory cell populations in the bone marrow and spleen and reduced the production of pro-inflammatory cytokine in monocytes and microglia. The mobilization of hematopoietic stem cells into the circulation was restored back to basal level after long-term pegfilgrastim treatment in SOD1 mice while the storage of Ly6C expressing monocytes in the bone marrow and spleen remained elevated. After pegfilgrastim treatment, an increased proportion of these cells in the degenerative muscle was detected at the end stage of ALS.

**Conclusions:**

GCSF attenuated inflammation in the CNS and the periphery in a mouse model of ALS and thereby delayed the progression of the disease. This mechanism of action targeting inflammation provides a new perspective of the usage of GCSF in the treatment of ALS.

## Background

ALS is a neurodegenerative disorder leading to progressive upper and lower motoneuron decline, muscle atrophy and death within a few years from diagnosis [1]. The majority of ALS cases are sporadic while up to 10% are familial. One of the mutations causing familial ALS occurs in the copper/zinc (Cu/Zn) superoxide dismutase 1 (SOD1) gene as first described by Rosen *et al*. [2]. SOD1 is a homodimeric metalloenzyme which catalyzes the conversion of a toxic superoxide anion O_2_^- ^to oxygen and hydrogen peroxide. The toxicity mechanisms of mutant SOD1 are not completely understood but mutant SOD1 is thought to cause the disease by a toxic gain of function which is linked to mutant SOD1 aggregation and oxidative stress [3]. Since the clinical and pathological nature of familial and sporadic ALS are similar, mechanisms of mutant SOD1 toxicity are thought to elucidate the disease mechanisms also in sporadic ALS. Transgenic animal models expressing mutant SOD1 have been widely used to study ALS pathogenesis [3].

ALS or SOD1-mediated pathology is accompanied by multiple cellular and subcellular abnormalities including deficits in the axonal transport and mitochondrial functions [3]. Abnormal SOD1 activity has also been shown to impair neuromuscular function [4,5]. Although the motoneuron degeneration is a hallmark of ALS [1] the disease may actually initiate from the periphery. Deficits in neuromuscular function may precede motoneuron deficits [6] and even the muscle-restricted expression of mutant SOD1 may be sufficient to initiate the ALS pathology with subsequent motoneuron degeneration [7]. Non-neuronal cells, namely microglia and astrocytes, participate in CNS homeostasis by secreting trophic molecules and helping to maintain proper neuronal signaling. On the other hand, they may also secrete molecules that disrupt the CNS homeostasis. Microglia and astrocytes thus play a crucial role in ALS pathology [8] by regulating neuroinflammation. Also, the microglia turnover by myeloid cells from the circulation may play a role in neurodegenerative diseases as previously reviewed [9]. In addition, peripherally derived myeloid cells may infiltrate not only into the CNS [10-13] but also migrate into the peripheral nervous system (PNS) or skeletal muscle [10,14] and potentially participate in ALS progression. Myeloid cells exist in different phenotypes, so-called pro-inflammatory and anti-inflammatory forms, also known as classical and alternatively activated monocytes, which may contribute to inflammation and tissue regeneration in a different manner [15]. The treatment modulating these monocyte subsets and thus inflammation may provide a potential therapy for neurodegenerative diseases.

GCSF is a hematopoietic growth factor which is currently in clinical use to mobilize stem cells into the circulation prior to apheresis [16] and to treat neutropenia after cytostatic therapy. GCSF has a wide variety of actions; it reduces apoptosis, drives neurogenesis and angiogenesis and attenuates inflammation [17-22]. GCSF is protective in myocardial infarction in animal models and it has also been tested for clinical use after acute and chronic ischemic heart diseases as reviewed by Kastrup *et al*. [23]. Moreover, GCSF has been shown to be protective in animal models of acute and chronic neurodegenerative diseases as reviewed in Diederich *et al*. [24], including stroke, Alzheimer's disease, Parkinson's disease and spinal cord injury [25-27]. GCSF was recently shown to be protective also in animal models of ALS [28], mediating its protective effects via P13/Akt pathway, an antiapoptotic transduction pathway downstream of GCSF signaling in neurons [17]. GCSF was also shown to be neuroprotective after peripheral axotomy [29,30].

GCSF is conventionally administered as repeated daily injections of filgrastim. Pegfilgrastim, a pegylated filgrastim, shows sustained activity *in vivo *because of its reduced renal clearance [31] which enables longer intervals for its application compared to filgrastim [32,33]. Peripherally administered GCSF is taken into the CNS most probably by a receptor-mediated transport [34]. In the CNS, GCSF receptors are expressed in neurons and microglia [17,28] but not astrocytes [17]. GCSF receptor is also expressed in peripheral monocytes and granulocytes [21,35]. In humans, GCSF administration increases the production of hematopoietic stem cells, granulocytes and monocytes [21]. GCSF therapy has been in phase I clinical trials for ALS where it has been proven to be a safe treatment for ALS [36-39]. However, the mechanism of action of GCSF is not fully known in ALS.

This study provides data for the usage of GCSF with sustained action in a mouse model of ALS. The long-term treatment with pegfilgrastim prolonged the survival of mutant SOD1 mice and attenuated both astro- and microgliosis in the spinal cord. Pegfilgrastim also modulated the inflammatory cell populations in the bone marrow (BM) and spleen in mutant SOD1 mice and reduced the production of pro-inflammatory cytokine TNFα. GCSF also reduced the inflammatory activation of microglia *in vitro*. After long-term pegfilgrastim treatment, the increased storage of Ly6C cells in the BM and spleen was accompanied by increased migration of monocytes, or their survival in the degenerative muscle at the symptomatic stage of ALS. This suggests that GCSF therapy delays the progression of ALS in a transgenic mouse model through the attenuation of inflammation in both the CNS and the periphery.

## Methods

### Animals

SOD1 G93A (B6. Cg-Tg-(SOD1-G93A)1Gur/J) transgenic mice carrying a high copy number of human mutant G93A SOD1 were obtained from Jackson Laboratory and maintained on C57BL/6J congenic background. SOD1 G93A mice manifest motor deficits between 17-19 weeks of age. Paralysis and end stage of the disease are reached by age of 24-26 weeks. Male SOD1 mice were used for survival and histological studies. Disease onset was determined by the wire-hang test. Each mouse was suspended while hanging from a wire cage top and latency was recorded. Deficits in motor performance were defined by the inability to hang for more than 3 minutes. The test was repeated 1-2 times per week until the mouse was sacrificed. For survival studies, the clinical end stage was defined as the inability of a mouse to right itself in a period of 30 seconds which was regarded as a criterion for mouse sacrifice. Animal experiments were conducted according to the national regulations of the usage and welfare of laboratory animals and approved by the Animal Experiment Committee in the State Provincial Office of Southern Finland.

### Pegfilgrastim treatment

A single injection of pegfilgrastim has been determined to equal to multiple injections of filgrastim in order to mobilize stem cells or treat neutropenia. We treated the mice with a submaximal dosage of pegfilgrastim as estimated from previous studies performed with wt mice [32,33]. Mice were treated with pegfilgrastim (Neulasta, Amgen) at the dosage of 300 μg/kg given subcutaneously once per week. Pegfilgrastim was diluted into 0.15 M sodium acetate (pH 7.4, adjusted with acetic acid) right before use. Sodium acetate injections were used as a vehicle control. The treatment was started at the age of 12 weeks for survival studies or at 12-16 weeks for histological studies. The treatment was continued once a week until the mouse was sacrificed. The littermates were randomly and equally divided into treatment groups. For histological studies, the pegfilgrastim-treated mice were sacrificed at the same age as when their littermates reached the clinical end stage.

### Immunohistochemistry

The mice were anesthetized with an overdose of tribromoethanol (Avertin, Sigma) and transcardially perfused with heparinized saline to remove blood from tissues. The meninges were removed from spinal cord which was then cut in half at the mid lumbar area and prepared as paraffin-embedded sections and cut with a microtome into 5 μm sections. The spinal cord sections were immunostained with microtubule associated protein 2 (Map2, Boehringer Mannheim), neuronal nuclear antigen (NeuN, Chemicon), choline acetyltransferase (ChAT, Chemicon), glial fibrillary acidic protein (GFAP, Chemicon) and ionized calcium binding adaptor molecule-1 (Iba-1, Wako), followed by the detection with a fluorescent or diaminobenzidine-based method as described [40]. The immunoreactive area was quantified from the ventral horn of the spinal cord. The total of 2 mm area of the mid lumbar spinal cord in each mouse was covered, analyzed as 20 sections 100 μm apart.

### Blood sample analysis

The blood sample was taken from the saphenous vein or from the heart at the time of sacrifice. Repeated blood sampling was avoided to keep the manipulation of hematopoietic system minimal. The blood sample was mixed with heparin and centrifuged to separate the blood cells. The plasma was collected and stored at -70°C until use. The GCSF concentration in blood was analyzed from plasma samples with a human GCSF ELISA kit (R&D Systems) according to manufacturer's protocol. The flow cytometry staining of blood cells was performed as described [40]. Briefly, nonspecific antigen binding was blocked with mouse IgG (clone MOPC-21, Sigma) and the cells were stained with fluorochrome-conjugated PE-CD117 (Stem Cell Technologies), PerCP-Gr-1 (BD Biosciences (BD)) and FITC-CD45 (BD), and analyzed on a FACS Calibur (BD) equipped with a single 488 nm argon laser. The data are shown as percentage of CD117 or Gr-1 cells of total CD45 blood leukocytes.

### Cell isolation and cell culture

Spinal cord neurons were obtained from E14 mouse embryos with a protocol modified from Vartiainen *et al*. [41]. Briefly, embryos were decapitated and the spinal cords were isolated. The meninges and the dorsal root ganglia were removed. Spinal cords were digested in 0.5 mg/ml papain (Sigma), 0.04 mg/ml DNAse in PBS 5-10 min at 37°C. Papain solution was replaced with 1 mg/ml BSA, 0.04 mg/ml DNAse, 10 mM glucose in PBS and gently triturated and centrifuged. The pellet was resuspended into DMEM, 10% FBS, 2 mM glutamine, penicillin-streptomycin (Gibco) and plated at 2.25 × 10^5 ^cells/cm^2 ^onto poly-D-ornithine-coated (Sigma) multiwell plates. The next day the medium was replaced with Neurobasal, L-glutamine, penicillin-streptomycin supplemented with B27 (all from Gibco), 25 μM glutamic acid (Sigma) and 10 μM AraC. After 24h, the medium was replaced with Neurobasal with supplements excluding AraC. One third of the medium was changed every three days and the cells were used for experiments after 9 days in culture. When preparing cultures from SOD1 mice, the spinal cord of each embryo was processed separately.

Mouse neonatal microglia cultures were prepared as described earlier [42,43]. BM cells were collected as described [40]. When needed, BM monocytes were obtained with the mouse monocyte negative enrichment kit (EasySep, Stem Cell Technologies) according to manufacturer's protocol. In splenocyte isolation, the spleen was first injected with HBSS, 2% FBS to balloon the spleen and the splenocytes were harvested by scraping the spleen gently with a needle to release the cells from the tissue. The cells were filtered through a 100 μm nylon mesh (BD) and centrifuged. The erythrocytes were lyzed with ammonium chloride as described [40].

Mononuclear cells were collected from thigh muscle and sciatic nerve with Percoll method as modified from Chiu *et al*. [14]. The tissue was minced with a scalpel and digested for 1 h at 37°C in 2.5 mg/ml collagenase D (Roche), 1 mg/ml DNAse (Roche) in HBSS (without divalent cations) and 10 mM HEPES (Gibco). During the isolation, samples were periodically vortexed gently. The homogenates were filtered through a 100 μm nylon mesh (BD) and centrifuged. The pellet was resuspended into isotonic 37% Percoll (Amersham) pH 7.4, in HBSS, 10 mM HEPES and carefully layered on top of 70% Percoll layer and centrifuged 500 g for 20 min at RT with no brake. Mononuclear cells were collected from the layer interphase.

When needed, the monocytes/mononuclear cells were cultivated in IMDM, 10% FBS, 2 mM L-glutamine and penicillin-streptomycin (reagents from Gibco, Invitrogen) in humidified atmosphere at 5% CO_2 _in 37°C. To enhance monocyte survival, the cells were incubated in the presence of 10 ng/ml MCSF (R&D Systems). Recombinant human GCSF (Peprotech) was used in cell culture studies instead of pegfilgrastim.

### Cell staining and flow cytometry

The staining of cells was performed as described [40]. Nonspecific staining was blocked with mouse IgG (MOPC-21, Sigma). Cells were stained with fluorochrome-conjugated FITC-CD3, FITC-CD45R, FITC-CD45, PE-CD11b, FITC-Ly6C, PerCP-Cy5.5-Ly6G (all from BD), PE-CD115 (Serotec), or CCR2 (Lifespan Technologies) followed by Alexa Fluor 488 (Molecular Probes). A minimum of 10 000 events were acquired on a FACSCalibur flow cytometer equipped with a 488 nm argon laser (BD) and data analysis was performed using Cellquest Pro software (BD).

### Quantitative real-time PCR

Spinal cords were homogenized for RNA isolation. The relative expression levels of specific inflammatory mediators tumor necrosis factor α (TNFα), inducible nitric oxide synthase (iNOS), nuclear factor erythroid 2-related factor 2 (Nrf2) target genes heme oxygenase 1 (HO1) and NADPH quinone oxidoreductase 1 (NQO1) were determined with quantitative RT-PCR as described [44]. The expression levels were normalized to ribosomal RNA and represented as fold change in the expression level of wt mice.

### Cytokine assay

Cells were treated with 10 ng/ml LPS for 24 h. Medium samples were collected and cytokine concentration determined with TNFα ELISA (R&D Systems). Detection of intracellular cytokine production was performed as described [45]. Briefly, cells were treated with 1 μg/ml LPS for 6 h including Brefeldin A (Sigma) for the last 4 h of incubation to inhibit protein transport and enhance the detection of intracellular cytokines. Cells were collected and stained for cell surface markers (Ly6C or CD11b) as described above. Cells were fixed with 4% paraformaldehyde for 20 min at RT then permeabilized with 0.05% saponin (Sigma). PE-conjugated TNFα, IL-6 or IL-10 cytokine antibody or an isotype control (all from eBioscience) was applied in PBS, 2% FBS, 0.05% saponin and incubated 30 min at RT. Cells were analyzed on a flow cytometer as described above.

### Nitric oxide assay

Cells were treated with 10 ng/ml LPS for 24 h in a phenol red-free medium. Medium samples were mixed with an equal volume of Griess reagent (2% phosphoric acid, 1% sulfanilamide, 0.1% naphthylethylene dihydrochloride, Sigma), incubated for 10 min and absorbance measured at 540 nm with a multiscan reader. Sodium nitrite (Sigma) was used as a standard.

### Cell viability assay

Cells were incubated in culture medium with 10 μM resazurin (Sigma) and incubated for 2 h (neurons) or 4h (monocytes). Medium samples were collected into a 96-well plate and measured by excitation at 544 nm and emission at 590 nm on a multiplate reader (Victor Wallac).

### Western blot

PAkt was analyzed from cell culture samples with western blotting as described [46].

### Statistical analysis

The data are expressed as mean ± SD. The data were analyzed with SPSS software using Kaplan-Meier survival statistics with a log rank sum test for testing differences in mouse survival, and using general linear model for testing differences in the motor performance test. Other data were analyzed with Student's T-test, Mann-Whitney U-test or one-way Analysis of variance (ANOVA) when appropriate, followed by Dunnett's or Tukey's *post hoc *test. * (p <0.05), ** (p <0.01), *** (p <0.001).

## Results

### GCSF with sustained activity prolongs the survival of SOD1 mice

We first determined the plasma concentrations of GCSF in mutant SOD1 mice after administration of pegfilgrastim. After an injection of pegfilgrastim, the plasma concentration of GCSF remained elevated for several days (1A). However, the plasma concentration of GCSF lowered to basal level before the next dosage, given at one week intervals. The data is shown after long-term administration of pegfilgrastim (1A, black squares), indicating that a prolonged application of a growth factor peptide was not hampered by a formation of neutralizing antibodies, a possible risk linked to cytokine or growth factor [47-50] therapy. This is further confirmed by the fact that the first dosage of pegfilgrastim (1A, open squares), analyzed at days 1 and 4, displayed similar GCSF rise and decay in plasma as observed after the prolonged pegfilgrastim therapy. The classic effect of GCSF was detected by increased neutrophil and stem cell counts in the peripheral blood (1B and 1C, p <0.01) after the first dose of GCSF. However, the leukocytosis was restored back to the basal level after prolonged pegfilgrastim treatment. Despite sustained plasma levels of GCSF after long-term therapy, the excess mobilization of leukocytes into the circulation was most likely hindered due to a compensatory mechanism.

**Figure 1:**
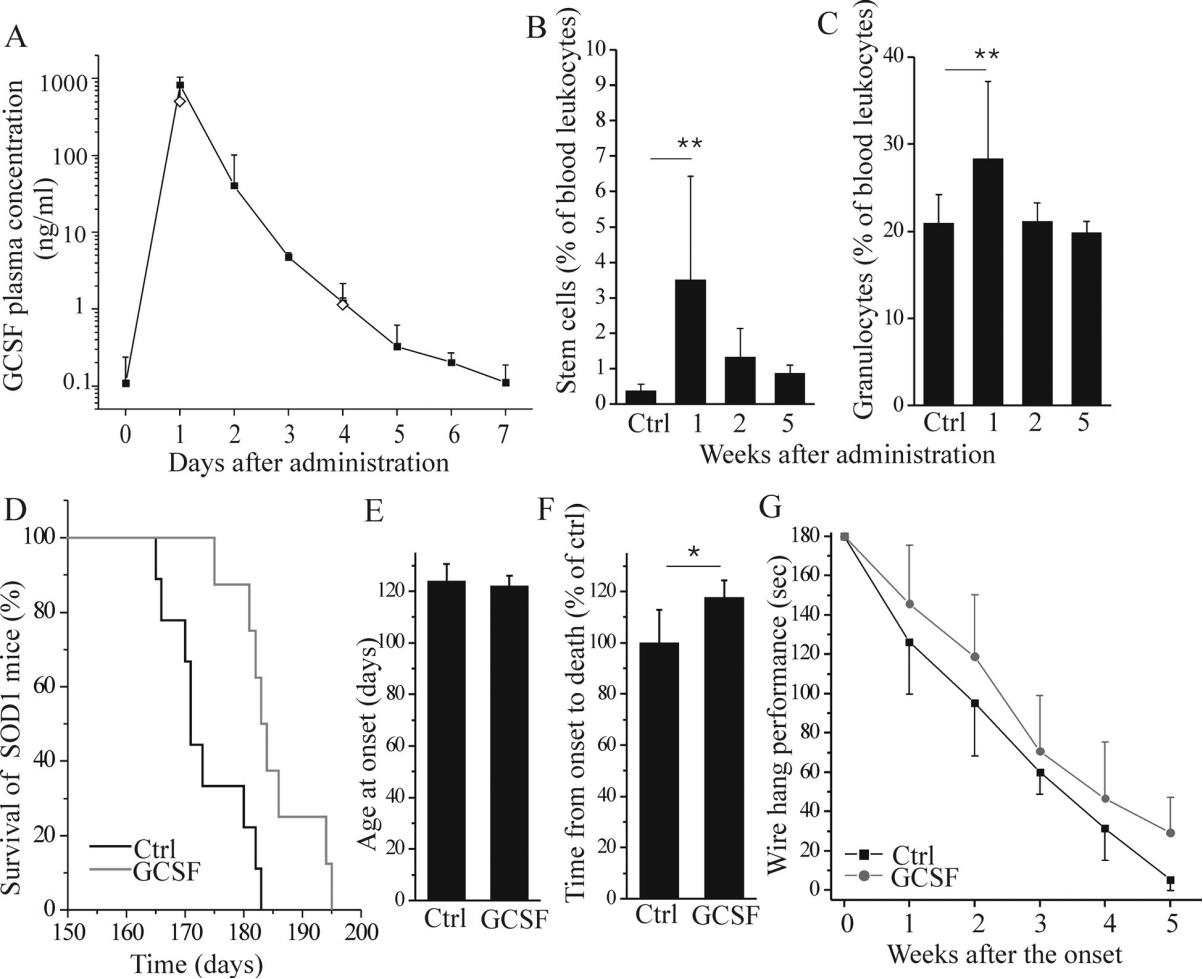
**GCSF with sustained activity has biological efficacy and prolongs the survival of mutant SOD1 mice**. Mutant SOD1 mice were treated with pegfilgrastim once a week starting at the age of 12 weeks. Plasma concentration of GCSF remained elevated multiple days after a single injection of 300 μg/kg (A) but returned back to baseline at day 7 before the next dosage (data was obtained from 33 mice, 1-2 blood samples/mouse). This occurred in a similar manner after the first dosage (A, open squares) as well as after the long-term pegfilgrastim treatment (A, black squares). Pegfilgrastim elevated the levels of stem cells (B, p <0.01, n = 11) and granulocytes (C, p <0.01, n = 11) after the first dosage, as analyzed four days after the pegfilgrastim administration, but the mobilization was decreased after prolonged pegfilgrastim treatment (n = 5). Long-term pegfilgrastim treatment increased the survival of mutant SOD1 mice as shown with a Kaplan-Meier survival graph (D, p <0.01, n = 8-9). Pegfilgrastim treatment did not delay the onset of the disease as determined by wire-hang test (E, n = 6) but increased the time from the onset to death (F, p <0.05, n = 4-5). The prolonged survival was accompanied with improved motoric performance in wire-hang test (G, p <0.05, n = 6) when analyzed within 5 weeks after the onset. The first mice died 5 weeks after the onset.

Prolonged pegfilgrastim therapy increased the survival of SOD1 mice (1D, p <0.01). When compared to their vehicle-controlled littermates, pegfilgrastim increased the life expectancy from 173 ± 6.7 days to 185 ± 6.7 days with the range of 3-23 days. The time of onset did not differ between the groups (1E) and the time from the onset to death was thus prolonged with the pegfilgrastim treatment (1F, p <0.05). The increased survival time was accompanied by improved performance in a wire-hang behavioral test (1G, p <0.05) indicating more sustained motoric capacity in GCSF-treated mutant SOD1 mice compared to vehicle-treated littermates.

### GCSF conducts neuroprotection *in vitro*

In order to uncover cellular and molecular mechanisms of GCSF-mediated neuroprotection we tested the action of GCSF on primary neuronal culture. Spinal cord neuron culture consisted of NeuN positive neurons and to a lesser extent, SMI-32 positive motoneurons, and the latter defined by the large size of the cell body and the long axon typical to motoneurons [51] in spinal cord cultures (2A and 2B). On non-stimulated neuronal cultures, the treatment with GCSF increased the Akt phosphorylation (2C, p <0.05) as shown earlier in NSC34 secondary cells [28]. When spinal cord neurons were exposed to glutamate-induced excitotoxicity *in vitro*, GCSF reduced neuronal cell death (2D, p <0.001). GCSF did not have any effect on neuron survival in control conditions (2D). When spinal cord neuron culture was prepared from mutant SOD1 mice we discovered that the general cell viability was slightly reduced in mutant SOD1 neurons and GCSF could alleviate the compromised cell viability (2E, p <0.05). However, unlike wt cells, GCSF did not protect mutant SOD1 neurons from glutamate neurotoxicity (data not shown).

**Figure 2:**
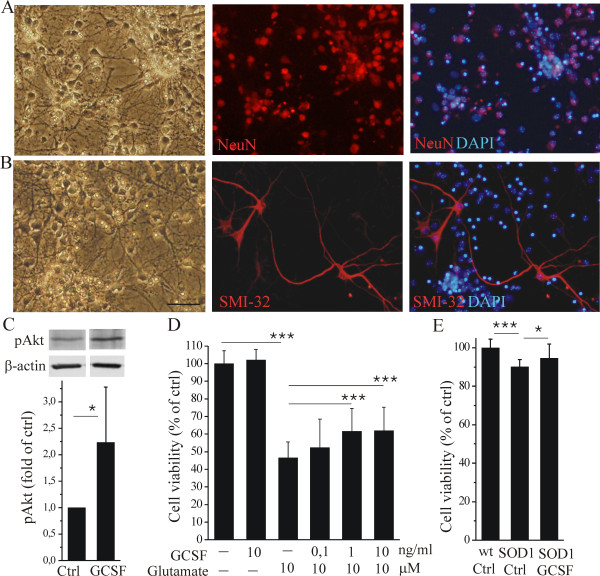
**GCSF is neuroprotective *in vitro***. Primary spinal cord neuronal culture expressed >90% neurons, majority of which were interneurons, positive for NeuN (A) while a small fraction of cells were motoneurons, positive for SMI-32 (B). When spinal cord neurons were treated with GCSF for 24 h, the Akt phosphorylation (pAkt) was increased (C). β-actin is shown as a loading control and the data was normalized to β-actin control in quantification. GCSF increased pAkt as quantified from western blot data (C, p <0.05, n = 3). The molecular weight of pAkt and β-actin are 60 and 42 kDa, respectively. When spinal cord neurons were exposed to glutamate, GCSF protected the neurons from glutamate excitotoxicity (D, p <0.001, n = 18-20). GCSF did not have any effect of neuron survival in control conditions (D, n = 14). When spinal cord neuron culture was prepared from mutant SOD1 mice, we discovered that the general cell viability was slightly reduced in mutant SOD1 neurons (E, p <0.001, n = 16 embryos). GCSF increased the cell viability in mutant SOD1 neurons (E, p <0.05, n = 16 embryos). The scale bar is 50 μm.

Next, to determine the effect of long-term pegfilgrastim treatment *in vivo*, spinal cord sections were analyzed by immunohistochemistry. The neurodegeneration was evident in mutant SOD1 mice when compared to wt mice, as evaluated by reduced immunoreactivity for neuronal markers (3A and 3B), Even though GCSF had certain neuroprotective properties *in vitro*, long-term pegfilgrastim treatment did not significantly increase spinal cord neuron survival in mutant SOD1 mice *in vivo *(3A and 3B).

**Figure 3:**
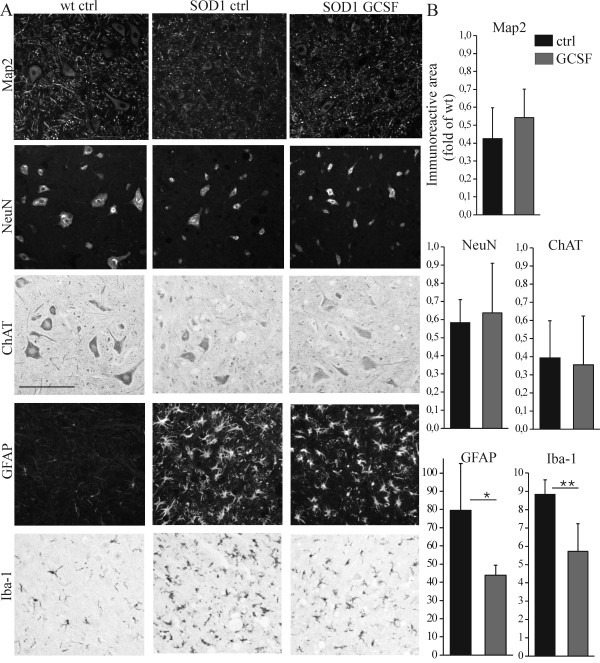
**GCSF with sustained activity attenuates inflammation in spinal cord in mutant SOD1 mice *in vivo***. Spinal cord sections were analyzed after long-term pegfilgrastim treatment from the end stage mutant SOD1 mice. The neuronal survival was decreased in mutant SOD1 mice compared to wt mice (A), determined from the ventral horn of the spinal cord. Long-term pegfilgrastim treatment did not notably rescue the cells from neurodegeneration in the spinal cord as determined with Map2, NeuN and ChAT immunostaining (3A and 3B, n = 5). However, when the inflammation status was analyzed from the same mice, long-term pegfilgrastim treatment decreased the inflammation in the spinal cord as determined with GFAP and Iba-1 immunostaining (A and B, p <0.05 and p <0.01 respectively, n = 5). The quantitation in (B) for Map2, NeuN, ChAT, GFAP and Iba-1 is shown as immunoreactive area in fold of wt mice spinal cord sections. The scale bar is 50 μm.

### GCSF with sustained activity attenuates inflammation *in vivo*

When the inflammation status was analyzed from the spinal cords of the same mice, long-term pegfilgrastim treatment indeed decreased astrogliosis and microgliosis in the ventral horn of the spinal cord as determined with GFAP (p <0.05) and Iba-1 (p <0.01) immunostaining, respectively (3A and 3B). We further examined whether reduction of inflammation in the spinal cord could be detected in the level of inflammatory mediators. Firstly, quantitative PCR results showed that the expression of TNFα was upregulated (34 ± 21 fold of wt, p <0.01) and that iNOS was downregulated (0.3 ± 0.1 fold of wt, p <0.05) in mutant SOD1 mice spinal cords compared to wt mice. Pegfilgrastim treatment decreased the expression of TNFα (to 19 ± 11 fold of wt) but interestingly, increased the expression of iNOS (to 0.8 ± 0.4 fold of wt), altering the inflammatory status closer to the wt situation, although these changes did not reach statistical significance. Since one of the downstream effectors of nitric oxide is Nrf2 [52-54], we further determined the Nrf2 target genes in the spinal cord. There was upregulation of HO1 and NQO1 in mutant SOD1 mice compared to wt mice (9.2 ± 1.5 fold of wt, p <0.001 and 2.9 ± 1.0 fold of wt, p <0.01, respectively) but pegfilgrastim did not affect the expression of these Nrf2 target genes. The expression of iNOS was thus not combined with Nrf2 target gene expression of HO1 and NQO1, but these were regulated independently. This also suggests that the protective effects of pegfilgrastim in mutant SOD1 mice were not mediated by Nrf2.

### GCSF attenuates inflammation in microglia and peripheral monocytes

Since TNFα was highly upregulated in the spinal cord of mutant SOD1 mice, we further determined the effect of GCSF on inflammatory cells in the CNS and peripheral hematopoietic organs. GCSF decreased inflammation-induced TNFα release in primary microglia cells *in vitro *(4A, p <0.001). GCSF also decreased the TNFα release in bone marrow (BM) monocytes *in vitro *obtained from wt and mutant SOD1 mice (4B, p <0.001). Furthermore, TNFα release was decreased in BM monocytes obtained from long-term pegfilgrastim-treated mutant SOD1 mice compared to vehicle-treated littermates (4C, p <0.05), suggesting a notable relevance *in vivo*. In addition to the reduction of TNFα production, the NO release was increased with GCSF in mutant SOD1 BM monocytes *in vitro *(4D, p <0.05) and in BM and spleen monocytes obtained from long-term pegfilgrastim-treated SOD1 mice (4E and 4F, p <0.001).

**Figure 4:**
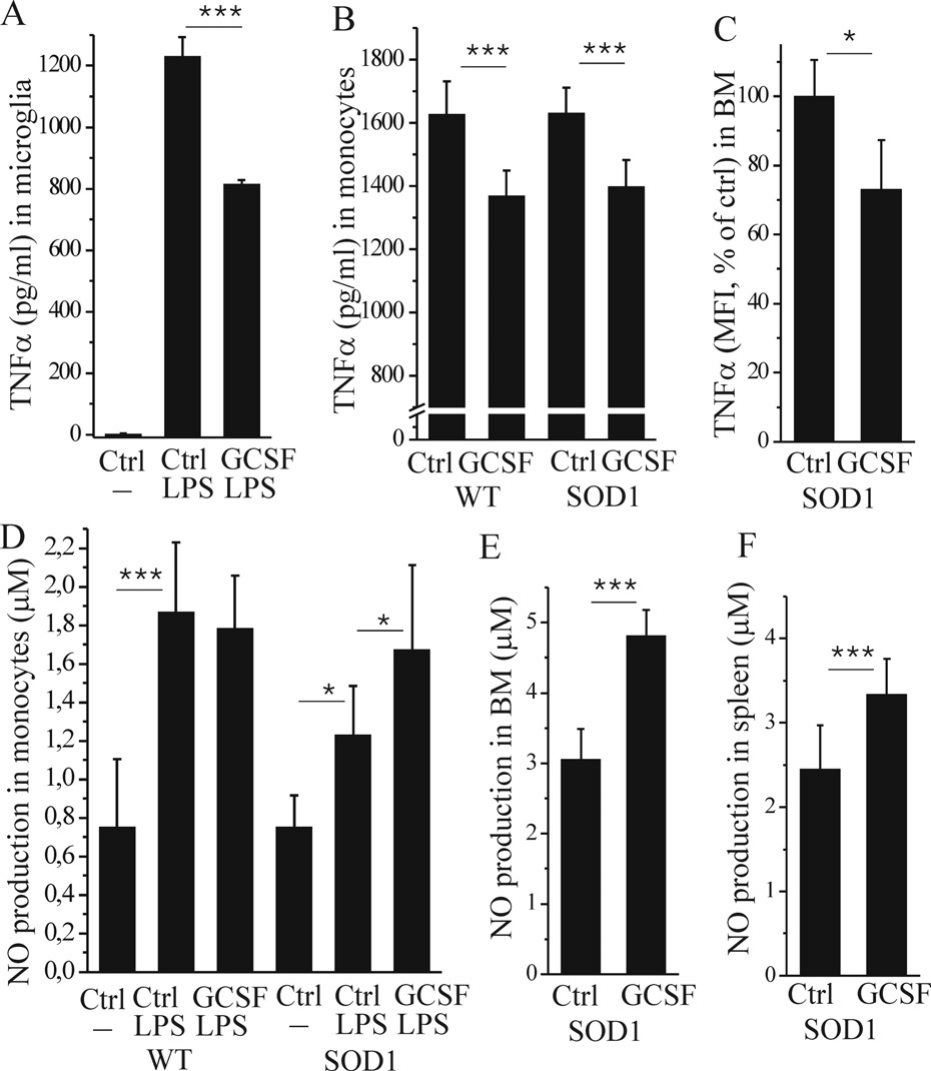
**GCSF modulates inflammatory responses *in vitro *and *ex vivo***. Cells were treated *in vitro *with 10 ng/ml LPS for 24 h and TNFα release was quantified with ELISA. GCSF decreased the production of TNFα in primary microglia (A, p <0.001, n = 3). GCSF also decreased the TNFα production in BM monocytes obtained from wt and mutant SOD1 mice (B, p <0.001, n = 6-7). TNFα production was also decreased in BM Ly6C monocytes isolated from 20 week-old, long-term pegfilgrastim-treated mutant SOD1 mice, as compared to their vehicle-treated littermates (C, p <0.001, n = 3): TNFα production was analyzed with intracellular cytokine staining with flow cytometry after 4 h incubation of 1 μg/ml LPS *ex vivo*. NO production was detected with determination of NO metabolites NO_2 _and NO_3 _from the medium after 24 h of incubation with 10 ng/ml LPS. NO production was increased after LPS stimulation in wt (D, p <0.001, n = 4-8) and mutant SOD1 BM monocytes *in vitro *(D, p <0.05, n = 7-8). GCSF further increased the NO production in mutant SOD1 monocytes up to the level of wt monocytes (D, p <0.05, n = 7-8). NO production was also increased in BM (E, p <0.001, n = 3) and spleen (F, p <0.001, n = 3) leukocytes obtained from long-term pegfilgrastim-treated mutant SOD1 mice, as compared to their vehicle-treated littermates: NO production was analyzed after 24 h of incubation with 100 ng/ml LPS.

### GCSF modulates cell populations in peripheral hematopoietic organs and in degenerating muscle

When measuring spleen size, we discovered decreases in weight (5A, p <0.01) and length (5B, p <0.001) of end stage mutant SOD1 mice compared to wt controls at the same age. In comparison, the body weight of mutant SOD1 mice at the end stage had dropped to 75 ± 14% of wt mouse weight. Pegfilgrastim treatment increased the spleen size of mutant SOD1 mice (5A and 5B, p <0.001) in a similar manner to wt mice indicating a long-term biological efficacy of GCSF on SOD1 mice. We further analyzed hematopoietic cell populations after long-term pegfilgrastim treatment from 20-week old mutant SOD1 mice. GCSF increased the number of total splenocytes (5C, p <0.001) but not the total number of BM leukocytes (5D). In BM, the number of lymphocytes was either not affected (CD3; 0.5 ± 0.1 million in control (vehicle-treated) and 0.4 ± 0.1 million in GCSF-treated) or decreased (CD45R; 5.7 ± 0.8 million in control and 1.7 ± 0.3 million in GCSF-treated, p <0.001) in response to pegfilgrastim treatment. On contrary to this, the number of Ly6C monocytes was increased (5E, p <0.01) in BM after pegfilgrastim treatment. Notably, the number of Ly6C^int ^cells was increased. Ly6C is a marker for myeloid lineage of cells and a component of myeloid differentiation antigen Ly6C/Ly6G, the latter expressed mainly in polymorphonuclear lineage of myeloid cells. Ly6C cells also expressed CD11b, an integrin involved in adhesive interactions and migration of myeloid cells. The number of cells expressing CD115, the receptor for macrophage colony stimulating factor was however not increased by pegfilgrastim.

**Figure 5:**
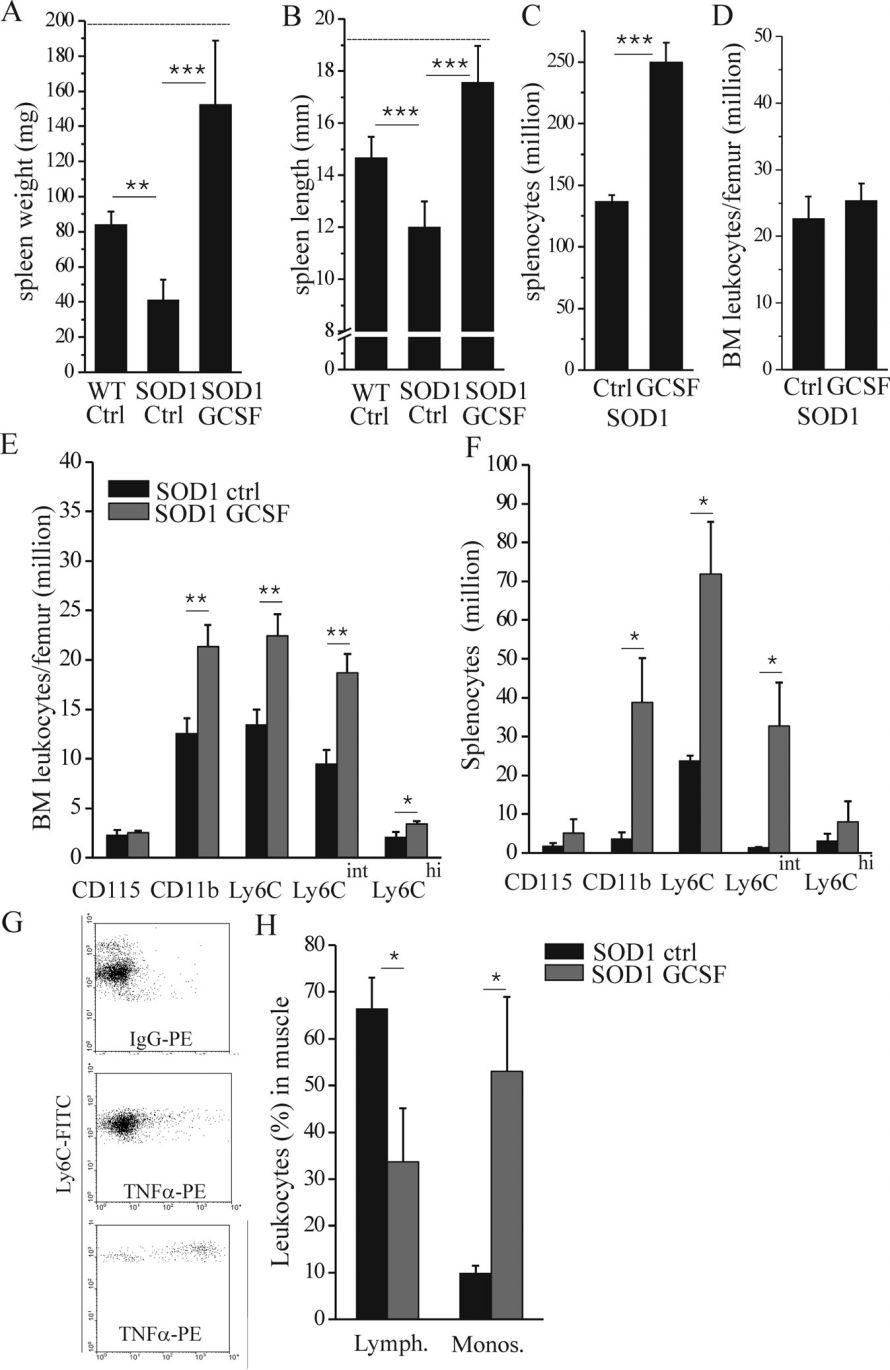
**GCSF with sustained action increases the availability of monocytes in hematopoietic organs and monocyte recruitment into the degenerating muscle**. Spleen size was decreased at late stage mutant SOD1 mice as assessed by weight (A, p <0.01, n = 6-11) and length (B, p <0.001, n = 6-11) as compared to wt control mice at the same age. Pegfilgrastim treatment increased the spleen size of mutant SOD1 mice (A, B, p <0.001, n = 6-11). The dotted line represents the spleen size of wt mice after long-term pegfilgrastim treatment (A, B). Long-term pegfilgrastim treatment also increased the number of splenocytes (C, p <0.001, n = 3) as analyzed from 20-week old mutant SOD1 mice. The number of leukocytes in BM was determined from the same mice (D): pegfilgrastim did not affect the total leukocyte number in BM but altered the composition of BM cell populations increasing the number of monocytes (E, p <0.01, n = 3). The similar effect on cell composition was detected is spleen cell populations (F, p <0.05, n = 3). The monocyte population which number was greatly increased, namely Ly6C^int ^had low or no production of cytokine after LPS stimulus (G, middle), in comparison to Ly6C^hi ^monocytes which represented high cytokine production for inflammatory stimulus (G, below) as analyzed by flow cytometry. When mononuclear cell population was analyzed from the thigh muscle including the sciatic nerve in mutant SOD1 mice at symptomatic stage, the Ly6C monocytosis was increased while lymphocytosis was decreased (H, p <0.05, n = 3).

In spleen, the number of lymphocytes was elevated (CD3; 31 ± 1.6 million in control and 44 ± 3.5 million in GCSF-treated, p <0.01) or remained at the same level (CD45R; 86 ± 8 million in control and 106 ± 32 million in GCSF-treated) in response to pegfilgrastim treatment. Similar to BM, the number of monocytes was increased (5E, p <0.05) in spleen after long-term pegfilgrastim treatment. Particularly the number of Ly6^int ^population of monocytes which represented low or no pro-inflammatory cytokine activation in response to an inflammatory stimulus was elevated (5E, 5F, 5G). These findings thus indicate that pegfilgrastim increased the production/storage of Ly6C monocytes in BM and spleen.

When mononuclear cell population was analyzed from the thigh muscle including the sciatic nerve at the symptomatic stage of ALS when there are pronounced deficits of motoric function, the Ly6C monocyte number was increased while lymphocyte number was decreased in response to pegfilgrastim treatment (5H, p <0.05). Lymphocytes were distinguished from monocytes based on their side scatter and forward scatter properties and their expression of lymphocyte or monocyte markers as defined above. GCSF probably increases the storage of certain Ly6C monocytes with migratory properties in spleen and BM. GCSF was also detected to increase the number of CCR2 expressing cells, which is a marker for migratory cells, in BM and spleen as analyzed with flow cytometry (data not shown). In summary, this suggests the GCSF treatment increased the availability of monocytes in the symptomatic stage of ALS in SOD1 mice. Since these cells have a favorable inflammatory profile, they may be recruited to the degenerative muscle as an attempt for recovery processes.

## Discussion

GCSF has proven to be a safe treatment in ALS in phase I studies, administered as a single [36,37] or repeated cycles with three months interval [38,39]. GCSF mobilizes hematopoietic stem cells from ALS patients in a consistent manner [55] and GCSF-mobilized stem cells could be transplanted back to ALS patients causing no adverse effects [36]. The clinical trials so far have shown limited improvement in ALS pathology. The reason for this may be the fact that GCSF was administered for a short period of time; only single or repeated cycles of few days of duration were given. Since long-term usage of GCSF may have severe side effects such as splenomegaly and even a spleen rupture, the targeted GCSF delivery may be an option if the mechanisms of action of GCSF were known in detail. The intraspinal delivery of GCSF with an adeno-associated virus (AAV)-GCSF vector was beneficial in mutant SOD1 mice; the neuromuscular junctions were preserved and motoneuron survival was increased [56]. Although the local intraspinal or intramuscular delivery of AAV-GCSF, it still had a systemic effect with increased levels of GCSF in serum and the induction of hematopoiesis [56]. Even though GCSF has a moderate effect on mutant SOD1 mouse survival, as we describe in this study, the GCSF-mediated effects on the modulation of inflammation are interesting and highly relevant in the light of ALS pathology.

In animal studies, long-term GCSF (filgrastim) administration with a subcutaneously implanted osmotic minipump, has been shown to increase the survival of mutant SOD1 mice accompanied with increased motoneuron survival and induction of an anti-apoptotic pathway in neurons [28]. Also, the GCSF expression restricted to CNS enhanced mutant SOD1 mouse survival. The mechanism of GCSF effect was suggested as a direct protection of motoneurons. The increase in the motoneuron survival was detected when analyzed at 15 weeks of age, *i.e*. at the time of clinical onset and no effect on astro-or microgliosis was detected when analyzed at 19 weeks of age. Using the same mutant SOD1 strain in our studies we detected an increase in the survival of mutant SOD1 mice after long-term administration of GCSF with weekly injections of pegfilgrastim. This pegylated filgrastim has a sustained effect due to the reduced renal clearance. We suggest pegfilgrastim to slow the progression of the disease since the treatment did not affect the onset of the disease. However, it is rather unlikely that pegfilgrastim treatment started at the age of 12 weeks could still postpone the onset of motor deficits since subclinical pathological features of ALS have already progressed at the time of clinical onset at around 17 weeks when first motor deficits can be detected in SOD1 mouse. When analyzed at the end stage of ALS, we did not detect neuroprotection in spinal cord of mutant SOD1 mice to same extent as detected in younger, symptomatic SOD1 mice [28]. However, we detected an evident reduction in astro- and microgliosis in the spinal cord of pegfilgrastim treated mutant SOD1 mice, as analyzed with GFAP and Iba-1 immunostaining reacting with astrocytes and all monocytic cells in the spinal cord, respectively. When studied with primary spinal cord neurons *in vitro*, we detected a GCSF-mediated neuroprotection with a similar activation of the P13K/Akt anti-apoptotic pathway as described earlier [28]. However, our results suggest that the anti-inflammatory effect of GCSF plays an important role in the progression of ALS. Although GCSF was not able to significantly reduce the decline of neuronal density in the spinal cord when analyzed with immunohistochemistry at the end stage, it preserved the neuronal innervation in the periphery by preventing the presynaptic decline of neuromuscular transmission (Naumenko and Pollari *et al*., unpublished). This suggests that the reduction of inflammation by GCSF demonstrated in this study *in vitro *and *in vivo *is indeed accompanied with enhanced neuronal function.

Boillée *et al*. demonstrated that the mutant SOD1 expression in motoneurons is a determinant of the ALS onset while the expression of mutant SOD1 in macrophages/microglia participated in ALS progression in later stage [57]. In addition, the expression of mutant SOD1 only in motoneurons or astrocytes was not sufficient for ALS pathology [58,59]. However, mutant SOD1 expression in astrocytes caused a release of soluble factors toxic to motoneurons [60]. This suggests that myeloid cells/microglia and astrocytes, as inflammation participating cells have high input to ALS progression. In our study, the TNFα expression was increased over 30-fold in the spinal cord of mutant SOD1 mice compared to wt mice. This was accompanied by a pronounced astro- and microgliosis indicating inflammation. When we analyzed the inflammatory properties of mononuclear phagocytes, *i.e*. microglia and peripheral monocytes, we discovered that exogenously applied GCSF *in vitro *or pegfilgrastim treatment *in vivo*, respectively, reduces the capacity of TNFα production. Since the reduced production of TNFα was accompanied with increased but modest production of NO at the same cells, we suggest NO to conduct an advantageous signaling within this context. Although NO is generally linked to proinflammatory signaling, it may exert protective effect in attempt to combat for oxidative stress or increase anti-apoptotic signaling [61-64]. The long-term treatment with NOS inhibitor did not have a protection in mutant SOD1 mice which argues for the role of NO solely as a proinflammatory mediator in ALS [65]. NO was recently shown to be involved in GCSF-mediated regeneration in chronic myocardial disease [66].

When analyzed from the hematopoietic organs, pegfilgrastim treatment increased the number of monocytes with modest TNFα production capacity. These cells, which expressed Ly6C, are stored in hematopoietic organs and may be recruited into the degenerative tissue. Ly6C is expressed in migratory monocytes which may shuttle between the circulation and the BM and enter the inflammatory tissue to become dendritic cells or macrophages [67-71], which participate in inflammatory and healing processes. Recently, it was shown that in addition to BM, the spleen also works as storage for Ly6C monocytes which are released and transmigrated into the heart after myocardial infarction [70]. We detected pegfilgrastim treatment to increase the availability of Ly6C monocytes in both BM and spleen. We also found that pegfilgrastim increased the proportion of monocytes out of other leukocytes in the muscle in mutant SOD1 mice when analyzed at symptomatic stage. This finding suggests that the increased availability of monocytes in the hematopoietic organs may also increase the availability of regenerative monocytes in the damaged tissue.

Furthermore, since BM cells have been shown to migrate into the spinal cord of mutant SOD1 mice [10-13] and peripheral nerves/muscles [10,14], the increased availability of the migratory subpopulation of monocytes which have reduced proinflammatory activation may decrease the inflammation and neurotoxicity and on the other hand, enhance the regeneration processes [72,73]. As recently demonstrated, the mutant SOD1 mice accumulated macrophages from the early stage of ALS throughout the disease progression [14,74]; the spinal cord microglia were endogenously-derived while in the PNS the majority of macrophages were originated from the circulation. This further emphasizes the importance of GCSF effect on the increased availability of migratory monocytes with reduced proinflammatory action.

The induction of motoneuron death and neurodegeneration in ALS has been described as a "dying forward" manner where the motoneuronal pathology starts from at the soma [75], or as a "dying back" manner where the motoneuronal pathology starts at the distal axons and proceeds back to the motoneuron soma [6]. Since GCSF exerts its inflammation-modulating effects in the CNS and the periphery, it may hinder the disease progression at multiple sites. In our study, GCSF strongly modulated the composition of inflammatory cell populations, their availability and potentially also their migration into degenerative muscle in a mouse model of ALS. It remains to be further investigated whether i) the transplantation of BM or spleen cells obtained from GCSF-mobilized mouse or ii) the transplantation of *ex vivo *GCSF-treated monocytes is sufficient to achieve the GCSF-mediated protection in a mouse model of ALS and which monocyte subpopulations in particular are involved in these processes.

## Conclusions

The present data demonstrate that GCSF attenuates inflammation in a mouse model of ALS which slows down the progression of the disease. GCSF reduced inflammation in the CNS and the periphery while increasing the availability of anti-inflammatory migratory monocytes. This mechanism of action targeting neuroinflammation and peripheral inflammation-participative cells provides a new perspective of the usage of GCSF in the treatment of ALS.

## Competing interests

The authors declare that they have no competing interests.

## Authors' contributions

EP conducted *in vivo *and *in vitro *studies. ES, TM, GG and TA participated in *in vivo *studies. ES, MJ, KK, SW and RG participated in *in vitro *studies. JM performed *in vitro *studies and wrote the manuscript. EP, JK and JM designed the study. EP, SW, RG and JK revised the manuscript. All authors have read and approved the final manuscript.
